# A Randomized Controlled Trial of an Employment Program for Veterans Transitioning from the Military: Two-Year Outcomes

**DOI:** 10.1007/s10488-022-01208-z

**Published:** 2022-07-12

**Authors:** Gary R. Bond, Monirah Al-Abdulmunem, Daniel R. Ressler, Daniel M. Gade, Robert E. Drake

**Affiliations:** 1 Social Policy and Economics Research, Westat, Rivermill Commercial Center, 85 Mechanic St., Lebanon, NH 03766 USA; 2 Virginia Department of Veterans Services, Richmond, VA USA

**Keywords:** Military veterans, Employment, Career mentoring, Veteran transition services

## Abstract

**Background:**

Military personnel face numerous challenges transitioning from military jobs to meaningful civilian employment. Many veterans seek help finding employment, but few veteran employment programs have been rigorously studied. Transitioning veterans generally have access to Local Community Resources (LCR), which include the Veterans Health Administration vocational rehabilitation services, the state-federal Vocational Rehabilitation program, and the Department of Labor’s American Job Centers. By contrast, the innovative National Career Coach Program (NCCP) offers intensive career coaching and financial incentives for working.

**Methods:**

This study used a randomized controlled design to compare the NCCP and LCR approaches for 208 transitioning service members (recent or pending transition). Researchers conducted interviews by telephone every four months for two years. Outcomes included earnings, months worked, and standardized self-report measures of health and well-being.

Findings

At two-year follow-up, significantly more NCCP participants had worked in paid employment than LCR participants (95% vs. 83%). NCCP participants averaged $2568 in monthly earnings compared to $1865 for LCR participants, thus averaging $16,872 more total income per participant over the two-year period. Employment outcomes significantly improved between Year 1 and Year 2. NCCP participants also reported significantly greater improvements in both physical and mental health compared to LCR participants. Average monthly earnings correlated with changes in health outcomes.

**Conclusions:**

Veterans receiving multi-faceted employment services early in the transition from the military showed sustained benefit over a two-year period with increased earnings over time and improved mental and physical outcomes. Positive employment outcomes may have contributed to improved health outcomes.

**Supplementary Information:**

The online version contains supplementary material available at 10.1007/s10488-022-01208-z.

## Introduction

Since 2003, over 200,000 enlisted men and women have separated annually from the U.S. military (GAO, 2019; Zogas, 2017). The transition from the military is difficult for many veterans, especially for those serving in the post-9/11 era (i.e., military service after September 2001) (Aronson et al., 2019; Dexter, 2020; Keeling et al., 2018; Mobbs & Bonanno, 2018; Prudential, 2012; Sherman et al., 2015). Most veterans experience significant mental and physical health symptoms during this transition period (Blore et al., 2015; Chandrasekaran, 2014; McNally & Frueh, 2013; Oster et al., 2017; Vogt et al., 2020). Starting a new job in the civilian labor force may be the swiftest means for a successful transition to civilian life (Stern, 2017). Employment provides daily structure, sense of purpose, and social connectedness. Yet for many veterans, the biggest transition challenge is finding employment that matches their skills and interests (Castro & Kintzle, 2017; Castro et al., 2013; Loughran, 2014; Stern, 2017). Consequently, many transitioning veterans use outside help in the job-seeking process, most often accessing resources such as online job banks, resume preparation services, and job fairs (Perkins et al., 2020; Zogas, 2017). But veterans report limited success with online hiring initiatives, suggesting the need for more intensive employment programs (Zogas, 2017). A much smaller percentage of veterans receive help through more intensive programs administered by a variety of community, government, private, and faith-based organizations. However, rigorous evaluations of employment programs for veterans are rare (Collins et al., 2014).

Among employment programs available to veterans, only the Individual Placement and Support (IPS) model of supported employment has been shown to improve employment outcomes (Davis et al., 2018a, 2018b; LePage et al., 2020; Ottomanelli et al., 2014; Resnick & Rosenheck, 2007). However, veterans have limited access to IPS, which is mostly available in medical centers, and IPS programs are mostly restricted to veterans with diagnosed serious psychiatric or physical disabilities (Abraham et al., 2014). Many veterans, especially those with service-connected disabilities, need employment assistance.

To address the multifaceted employment challenges facing transitioning veterans, we designed and implemented a new employment program for this study. NCCP is a national program that incorporates specific components associated with better employment outcomes in a large-scale longitudinal study of transitioning veterans (i.e., career planning, resume development, networking, interview practice, and translating military experience into civilian job requirements) (Perkins et al., 2021; Vogt et al., 2018). Our preliminary report on a randomized controlled trial examining the NCCP showed that it improved employment outcomes over a one-year period (Bond et al., 2022b). The current paper examines the two-year findings for this evaluation. The current report also examines the impact of employment outcomes on health and well-being, a relationship previously documented in both the veteran and general populations (Carra et al., 2021; Drake & Wallach, 2020; Modini et al., 2016).

## Methods

### Overview

Independence Project was a randomized controlled trial comparing an intensive employment model (National Career Coach Program [NCCP]) to standard employment services (Local Community Resources [LCR]). The Westat Institutional Review Board approved the study, which followed the principles outlined in the Declaration of Helsinki.

### Interventions

NCCP has four components: a four-day, in-person, employment skills seminar held in Alpharetta, GA; up to 18 months of personalized job coaching provided by telephone and other remote contact; a human capital fund to pay for expenses of securing a job (e.g., travel, clothing, computers, professional organization fees); and an opportunity to earn a bonus for employment earnings (up to 25% of monthly earnings, capped at $825 a month). The program assigned an individual mentor/coach to each participant for the skills seminar and job coaching.

Participants assigned to LCR received an information sheet providing contact information for three local service providers offering training, financial assistance, and paid work experiences: the Veterans Health Administration vocational rehabilitation services, the state-federal Vocational Rehabilitation program, and the U.S. Department of Labor American Job Center.

### Sampling and Enrollment

The sample consisted of enlisted men and women transitioning from the military and seeking employment. Eligibility criteria included: under the age of 45, at least 6 months of active military service with an Honorable or General discharge, within 6 months before separation or 12 months after separation, either without civilian employment (before separation) or unemployed or working in short-term stopgap jobs (after separation), and receiving or applying for a service-connected disability rating from the Veterans Benefits Administration (VBA).

We recruited participants through letters, social media, online sources, and word of mouth. Using mailing lists from two data repositories maintained by the Departments of Veterans Affairs (VA), we sent recruitment letters to 28,000 recently discharged veterans. Online advertisements directed prospective respondents to a study website featuring a self-administered, qualifying survey (a series of screening questions that helped determine eligibility) and an invitation to those passing the screening questions to send contact information to the research team.

Trained interviewers conducted all research interviews by telephone. After determining that a prospect was eligible for the study, enrollment comprised four steps: informed consent, baseline interview, randomization, and connecting participants to their assigned program. Coaches assigned to NCCP participants contact them directly, while LCR participants received phone number for local community resource offices.

### Measures

#### Background Characteristics

We obtained detailed demographic information and details on military service, adapting questions from prior studies (Davis et al., 2018a, 2018b).

#### Employment Measures

We measured employment outcomes using the Dartmouth Vocational Update Form (Drake et al., 1996). The primary outcome (earnings from employment) included any paid employment, including income-generating self-employment. We also measured competitive employment, defined as permanent community jobs, which excludes sporadic employment in the gig economy (such as an Uber driver). We used the interview data to construct a 24-month timeline of job starts and endings to determine monthly employment status, earnings, number of months worked, and time to first job. We assessed job satisfaction using a single-item global satisfaction measure, which has been associated with job retention (Resnick & Bond, 2001), and a single-item satisfaction with vocational services measure (Kukla & Bond, 2009). Two-year job satisfaction was calculated as the mean of job satisfaction ratings made during the 16-, 20-, and 24-month interviews.

#### Service-Connected Disability

The VBA awards financial compensation to veterans who have service-connected disabilities (Volberding et al., 2019). The amount of compensation, determined by the veteran’s disability rating, ranges from 0 to 100% based on an aggregated score for one or more physical and mental conditions. Veterans may apply for adjusted ratings over time. When first interviewed, participants were in various stages of applying for and receiving a disability rating; we therefore asked participants to provide this information over the course of the baseline and subsequent interviews.

#### Satisfaction with Life Scale

This 5-item scale is a widely used self-report scale to measure life satisfaction (Diener et al., 1985). The scale has good convergent and discriminant validity and temporal stability (Pavot & Diener, 1993). The internal consistency coefficient (Cronbach’s alpha) in the study sample was 0.85.

#### Veterans Rand-12 (VR-12)

This measure, a slight modification of the well-validated SF-12 (Ware et al., 1996), is a 12-item self-reported assessment of health widely used in veteran populations (Selim et al., 2009). The VR-12 yields two subscale scores, the Mental Component Score (MCS) and the Physical Component Score (PCS). The VR-12 is psychometrically valid, with good internal consistency, test–retest reliability, and criterion-oriented validity (Salyers et al., 2000). The internal consistency coefficients (Cronbach’s alpha) for MCS and PCS were 0.89 and 0.85, respectively, in the study sample.

#### Patient Health Questionnaire-9 (PHQ-9)

The PHQ-9 is a 9-item self-report depression checklist that has been well validated in two large studies and has been used in many medical surveys (Kroenke & Spitzer, 2002). The internal consistency coefficient (Cronbach’s alpha) for the PHQ-9 was 0.88 in the study sample.

#### InCharge Financial Distress Financial Wellbeing (IFDFW)

This 8-item checklist measures financial distress/financial security (Prawitz et al., 2006). The checklist has good psychometric properties, including content and construct validity and sensitivity to change (Garman et al., 2007). The internal consistency coefficient (Cronbach’s alpha) was 0.95 in the study sample.

#### Substance Use

Participants reported use in the last year of the following substances: tobacco, alcohol, marijuana and cannabis products, illegal drugs, and prescribed opioids. The interviewer then asked participants reporting substance use to indicate frequency of use.

### Statistical Analyses

We conducted exploratory data analysis on all relevant measures to determine their distributional properties (Tukey, 1977). Most analyses employed conventional univariate tests (t tests and chi-squares). For measures with skewed distributions (e.g., hourly wage), we made appropriate adjustments (e.g., using nonparametric tests or substituting log-transformed values for the measure in the statistical analyses). We examined group equivalence of NCCP to LCR through comparison of baseline characteristics. The main outcome analyses were endpoint analyses at 24 months using cumulative outcomes for employment measures and change measures for disability ratings and self-report measures of health and well-being. For all analyses, we used a significance level of p < 0.05 (two-tailed). We calculated effect sizes (*d*) for all between-group differences, using the standard formula for continuous measures (Cohen, 1988) and the arcsine transformation for dichotomous measures (Lipsey, 1990).

We assessed monthly employment rates using univariate tests of independent proportions. In addition, a multivariate test of the overall significance of the monthly employment rates using SAS—PROC GLIMMIX (Littell et al., 2006) assessed the group effect (NCCP vs. LCR), time effect (24 monthly observational periods), and group-by-time interactions. Similarly, we conducted two-way repeated measures analyses of variance on months worked and monthly earnings, examining the group effect (NCCP vs. LCR), time effect (Year 1 vs. Year 2), and group-by-time interactions.

The analyses of earnings data used the final analytic sample. The earnings analyses included earnings through the month of the last-completed interview. The research protocol includes follow-up interviews for a third year. As a result, these interviews occasionally permitted filling in missing objective employment records (including employment status, months worked, and earnings) for participants who did not complete the 24-month interview. We calculated the monthly earnings for the months for which we have employment records. We also examined employment outcomes in the worker sample, that is, the subgroup of participants who worked at least one day during the study period.

To test the hypothesis that employment outcomes were associated with health outcomes, we examined the Pearson correlations between two-year employment outcomes (number of months worked and monthly earnings) and two-year changes between baseline and two-year follow-up in health and well-being.

## Results

Between May 2018 and June 2019, we enrolled 229 participants, assigning 115 to NCCP and 114 to LCR. Of these, 21 were no-shows, that is, participants with whom we had no contact after baseline (10 NCCP, 11 LCR). The final analytic sample consisted of 105 (91.3%) NCCP participants and 103 (90.4%) LCR participants who completed at least one follow-up interview. Of these, 180 (87%) participants completed interviews at 12 months and 119 (57%) at 24 months. As shown in Table 1, we obtained employment outcomes for 184 (88%) participants at 12 months and 125 (60%) at 24 months. (Six participants who missed their 24-month interview competed later interviews.) Starting at 8 months, the attrition rate for the employment data was 8–15% higher for LCR compared to NCCP, a significant difference at each follow-up period. Over the first 20 months, the attrition rate was less than 20% for the NCCP sample but grew to 34% for the LCR sample.

**Table 1:** Employment data completion rates

	Completed (N, %)			Significance
	Total	NCCP	LCR	
Baseline	229	115	114	
No shows	21 (9%)	10 (9%)	11 (10%)	
Final analytic sample	208	105	103	
4-month	208 (100%)	105 (100%)	103 (100%)	–
8-month	198 (95%)	104 (99%)	94 (91%)	χ^2^ = 6.89, p = 0.01
12-month	184 (88%)	98 (93%)	86 (83%)	χ^2^ = 4.93, p = 0.03
16-month	165 (79%)	91 (87%)	74 (72%)	χ^2^ = 6.97, p = 0.01
20-month	153 (74%)	85 (81%)	68 (66%)	χ^2^ = 5.96, p < 0.05
24-month	125 (60%)	70 (67%)	55 (53%)	χ^2^ = 3.82, p = 0.05

As shown in Tables 2 and 3, 81% of the final analytic sample were men, 54% were under the age of 30, and 60% were married or cohabiting with a partner. All had at least a high school diploma or equivalent and 85% had postsecondary education experience; 55% identified as belonging to a racial minority and 19% as Hispanic. Participants lived in all regions of the U.S., including 64% in the Southern region. Study participants reported significantly poorer adjustment and greater distress on several standardized measures, including life satisfaction, mental health, symptoms of depression, and financial distress, compared to published norms in military and civilian (Bond et al., 2022a). As shown in Tables 2 and 3, with one minor exception, the two intervention groups did not differ at baseline on any demographic, economic, military experience, or health measures.

**Table 2:** Background characteristics of the final analytic sample

Characteristic	Total (n = 208)	NCCP (n = 105)	LCR (n = 103)	Test of significance
Female, n (%)	39 (18.8%)	17 (16.3%)	22 (21.4%)	χ^2^ = 0.85, p = 0.36
Age, M (SD)	30.49 (6.78)	30.51 (6.89)	30.47 (6.70)	t = 0.05, p = 0.96
Marital status, n (%)				χ^2^ = 0.93, p = 0.34
Married/Cohabiting partner	124 (59.6%)	66 (62.9%)	58 (56.3%)	
Unmarried	84 (40.4%)	39 (37.1%)	45 (43.7%)	
Education, n (%)				χ^2^ = 0.20, p = 0.66
High school diploma/GED	32 (15.4%)	15 (14.33%)2	17 (16.5%)	
Technical certificate and/or Higher education	176 (84.6%)	90 (85.7%)	86 (83.5%)	
Race, n (%)^a^				χ^2^ = 0.09, p = 0.77 (White vs. other)
White	93 (44.7%)	48 (45.7%)	45 (43.7%)	
Black or African-American	77 (37.0%)	41 (39.0%)	36 (35.0%)	
Asian	13 (6.3%)	4 (3.8%)	9 (8.7%)	
American Indian or Alaskan	2 (1.0%)	1 (1.0%)	1 (1.0%)	
Hawaiian/Pacific Islander	4 (1.9%)	1 (1.0%)	3 (2.9%)	
Other	32 (15.4%)	14 (13.3%)	18 (17.5%)	
Hispanic/Latinx Ethnicity	40 (19.2%)	22 (21.2%)	18 (17.5%)	χ^2^ = 0.45, p = 0.50

**Table 3:** Military characteristics and health and well-being ratings of the final analytic sample

Measure	Total (*n* = 208)	NCCP (*n* = 105)	LCR (*n* = 103)	Test of significance
Military branch, *n* (%)				χ^2^ = 0.58, p = 0.45 (Army vs. other)
Army	132 (63.5%)	64 (61.0%)	68 (66.0%)	
Air Force	28 (13.5%)	15 (14.3%)	13 (12.6%)	
Navy	27 (13.0%)	17 (16.2%)	10 (9.7%)	
Marine Corp	17 (8.2%)	8 (7.6%)	9 (8.7%)	
Coast Guard	4 (1.9%)	1 (1.0%)	3 (2.9%)	
On active duty at baseline	56 (26.9%)	27 (25.7%)	29 (28.2%)	χ^2^ = 0.16, p = 0.69
Served in a combat zone, *n* (%)	100 (48.1%)	53 (50.5%)	47 (45.6%)	χ^2^ = 0.49, p = 0.48
Years military service, M (SD)	8.70 (6.63)	8.88 (7.01)	8.52 (6.26)	t = 0.38, p = 0.70
SWLS^a^	4.54 (1.37)	4.61 (1.43)	4.48 (1.31)	t = 0.68, p = 0.50
VR-12^b^				
PCS	40.56 (11.01)	39.19 (11.29)	41.95 (10.59)	t = 1.82, p = 0.07
MCS	43.66 (14.38)	42.88 (13.50)	44.46 (15.25)	t = 0.79, p = 0.43
PHQ-9^c^	8.37 (6.18)	8.65 (6.49)	8.08 (5.85)	t = 0.67, p = 0.51
IFDFW^d^	5.38 (2.55)	5.77 (2.44)	4.98 (2.59)	t = 2.27, p = 0.02
Initial VBA Disability Rating	72.6% (22.8) (N = 172)	72.4% (22.7) (N = 95)	72.9% (23.2) (N = 77)	t = -0.12, p = 0.90

^a^Satisfaction with Life Scale: 1 = low satisfaction – 7 = high satisfaction

^b^PCS = Physical Component Summary; MCS = Mental Component Summary: Higher scores indicate better health

^c^*Patient Health Questionnaire-9:* Higher scores indicate more depression 1–4 Minimal depression while 20–27 Severe depression

^d^InCharge Financial Distress Financial Wellbeing Scale: 1: High distress/Low wellbeing—10: Low distress/High well-being

We compared the 24-month study completers (that is, the 125 participants with 24-month employment data) to no-shows (that is, the 21 participants who completed no interviews after baseline) and dropouts (the 81 participants with no 24-month employment data) on baseline characteristics (See Table A, Online Supplement). The completers were older, more likely married, better educated, more often on active duty at baseline, less likely to have a combat specialty in the military, and more likely to have served in a combat zone. The most robust statistical differences, however, were related to self-reported measures of health and well-being. Completers reported significantly greater satisfaction with life, less depression, and less financial distress than dropouts and no-shows at baseline. To check for group equivalence after attrition, we compared 24-Month NCCP completers to LCR completers on baseline characteristics. The two groups did not differ statistically on any characteristic (Results not shown).

### Service Use and Satisfaction

Most NCCP participants used each of the four NCCP components: 68% attended the in-person training, 98% had contact with their career coach, 72% used the human capital fund (mean amount awarded was $3187), and 78% of eligible participants received wage bonuses. Among those receiving bonuses, the cumulative amount averaged $8402. (The bonus was not included in earnings analysis below.)

A previous paper reported details of service use and satisfaction ratings from the first year of follow-up (Bond et al., 2022b), which we summarize here. Over the first year, 80% or more of NCCP participants interviewed were in contact with their career coach. The contact rate for interviewed NCCP participants remained high at 16 months (75%) and 20 months (84%) but dropped to 50% at 24 months. The large majority of NCCP participants who connected with NCCP reported satisfaction with the program at every follow-up interview (mostly over 90%).

By contrast, few LCR participants had contact with any of the three local resources they were offered. By the 4-month interview, 34% had contacted an American Job Center, 13% contacted a state vocational rehabilitation agency, and 20% had contacted VA vocational rehabilitation services. The contact rate decreased in subsequent months. Of those accessing local resources at 4 months, between 17 and 48% reported satisfaction with the services. In the second year, very few LCR participants had contact with any of the three employment programs.

### Employment Outcomes

Table 4 shows two-year employment outcomes for the final analytic sample. All four outcomes significantly favor NCCP over LCR. On average, NCCP participants earned $703 more per month than LCR participants ($2568 vs. $1865). Over a 24-month period, total difference in mean earnings was $16,872.

**Table 4:** Two-year employment outcomes for final analytic sample

	Total	NCCP	LCR	Test of significance	*d* effect size
	Mean (SD)	Mean (SD)	Mean (SD)		
	(*n* = 208)	(*n* = 105)	(*n* = 103)		
Worked in a paid job	185 (88.9%)	100 (95.2%)	85 (82.5%)	χ^2^ = 8.55, p < 0.01	0.41
Worked in a competitive job	175 (84.1%)	95 (90.5%)	80 (77.7%)	χ2 = 6.39, p < 0.02	0.32
Percentage of months in paid work	49.7% (32.6)	56.5% (30.2)	42.8% (33.7)	t = 3.08, p < 0.01	0.43
Monthly earnings	$2220 (2028)	$2568 (2073)	$1865 (1926)	t = 2.53, p < 0.05	0.36

**Fig. 1:**
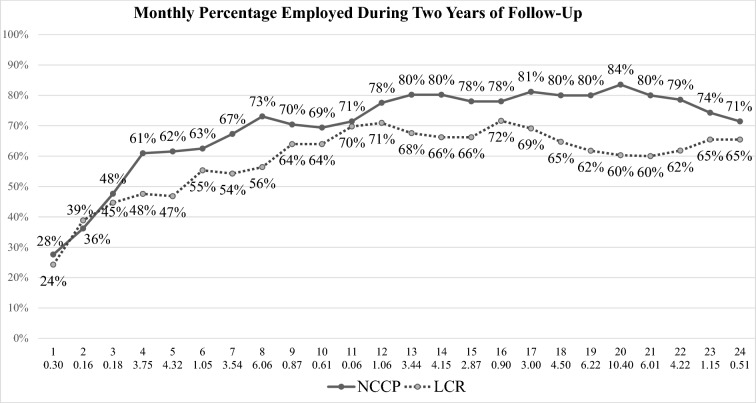
Monthly employment rates for NCCP and LCR. X axis indicates Month of Follow-up and chi-square value for difference between NCCP and LCR employment rates. Chi square values exceeding 3.84 are statistically significant at p < 0.05

Figure 1 shows the monthly employment rates over the 24-month period for the two groups. The monthly employment rate was significantly higher for NCCP than for LCR in 11 of the 24 monthly periods. The random effects logistical regression analysis found a significant time effect (the employment rate increased over time), *t* (4130) = 13.28, *p* < 0.001, and a significant interaction effect (over time, the increase in monthly employment was greater for NCCP than LCR), *t* (4130) = 2.82, *p* < 0.01. The main effect for study condition was not significant, *t* (4130) = 0.97. Among participants who worked during the follow-up, we found no differences between NCCP and LCR on time to first job, job satisfaction, highest hourly wage, or any other employment measure (Results not shown). The mean time to first job after baseline was 127 days (median = 84 days), mean (and median) job tenure in longest-held job was 335 days, and mean highest hourly wage was $23.68. Most participants who worked were full-time employees.

As shown in Table 5, two-way repeated measures analyses of variance found significant effects for group and year of follow-up for both months worked and monthly earnings. From Year 1 to Year 2 the mean annual earnings increased from $26,028 to $42,432 for NCCP, and from $19,620 to $30,528 for LCR. The difference in mean annual earnings between NCCP and LCR increased from $6408 (Year 1) to $11,904 (Year 2), though this increased gap in earnings (the group by time interaction) was not significant.

**Table 5:** Comparison on employment outcomes between groups over time

Year	NCCP (N = 91)	LCR (N = 74)	Two-factor repeated measures analysis of variance tests of significance		
			Group	Year	Interaction
	% of Months Worked				
	M (SD)	M (SD)			
Year 1	53.5% (32.1)	46.0% (35.0)	F (1,163) = 5.24, p < 0.05	F (1,163) = 24.35, p < 0.001	F (1,163) = 1.40, p = 0.24
Year 2	71.5% (37.1)	57.1% (40.1)			

	Monthly Earnings				
	M (SD)	M (SD)			
Year 1	$2169 (1905)	$1635 (1709)	F (1,163) = 5.81, p < 0.05	F (1,163) = 55.49, p < 0.001	F (1,163) = 2.24, p = 0.14
Year 2	$3536 (2695)	$2544 (2482)			

### Change in Health Measures from Baseline to 24 Months

Tables 6 and 7 show changes over two-year follow-up for five self-report measures of health and well-being. The NCCP group reported significantly greater improvements in both physical and mental health components of the VR-12 compared to the LCR group, who actually reported a worsening of health on both measures. Both groups reported a significant improvement in financial well-being. Both groups increased significantly in disability rating over time, but the two groups did not differ on 24-month disability rating or change in disability rating.

**Table 6:** Change in physical and mental health and well being from baseline to 24 months

	NCCP (N = 68)						LCR (N = 51)					
	Baseline		24 months		t value	p	Baseline		24 months		t value	p
	Mean	SD	Mean	SD			Mean	SD	Mean	SD		
SWLS^a^	4.72	1.42	4.71	1.41	0.04	0.97	4.59	1.25	4.74	1.48	− 0.83	.41
VR-12^b^												
PCS	40.04	11.38	43.80	10.91	− 3.29	0.002	42.22	11.13	40.55	10.59	1.03	.31
MCS	43.37	12.80	45.02	14.73	0.49	0.62	47.92	13.59	44.39	15.30	1.98	.05
PHQ-9^c^	8.10	6.27	7.59	5.52	0.88	0.39	6.61	5.04	7.29	6.06	− 0.94	.35
IFDFW^d^	6.04	2.52	7.24	2.57	− 4.18	< 0.01	5.41	2.91	6.85	2.64	− 4.68	< .01
Disability Rating^e^	71.6%	23.5	79.6%	21.7	4.56	< 0.001	76.8%	21.4	21.5	4.08	− 4.68	< .001

^a^Satisfaction with Life Scale: 1 = low satisfaction – 7 = high satisfaction

^b^PCS = Physical Component; MCS = Mental Component: Higher scores indicate better health

^c^Patient Health Questionnaire-9: Depression scores range from 1–4 (Minimal) to 20–27 (Severe)

^d^InCharge Financial Distress Financial Wellbeing Scale: 1: High distress/Low wellbeing—10: Low distress/High well-being

^e^VBA disability rating (Range 0%-100%). Comparisons are between initial and 24-month ratings. Sample size: NCCP (N = 67), LCR (N = 47)

**Table 7:** Change in physical and mental health and well being from baseline to 24 months

	Change scores (24 months—Baseline)						
	NCCP		LCR		t value	p	d
	Mean	SD	Mean	SD			
SWLS^a^	− 0.06	1.26	0.15	1.29	− 0.66	0.51	0.16
VR-12^a^							
PCS	3.76	9.44	− 1.67	11.56	2.82	0.01	0.51
MCS	1.65	11.95	− 3.53	12.74	2.27	0.03	0.42
PHQ-9^b^	− 0.51	4.85	0.69	5.22	− 1.29	0.20	0.24
IFDFW^a^	1.21	2.38	1.44	2.19	− 0.54	0.59	0.10
Disability Rating^b^	7.9%	14.2	6.4%	10.7	0.62	0.53	0.12

^a^Positive change score means improvement

^b^Negative change score means improvement

As shown in Table 8, both groups showed significant reductions in percentage of participants reporting prescription opioid use over two years (29% reduction for NCCP and 35% for LCR). The percentage of NCCP participants reporting tobacco use also significantly declined (13% reduction). Among 36 NCCP participants reporting alcohol use, the estimated number of alcoholic drinks per week declined from baseline (M = 8.4, SD = 9.8) to 24 months (M = 4.0, SD = 4.0), t = 2.92, p < 0.01.

**Table 8:** Change in percentage of participants using substances from baseline to 24 months

	NCCP (N = 68)			LCR (N = 51)			Percentage change	
	Baseline	24 months	McNemar's test	Baseline	24 months	McNemar's test		
	N (%)	N (%)	p	N (%)	N (%)	p	NCCP (%)	LCR (%)
Tobacco	22 (32.4%)	13 (19.1%)	0.04	17 (33.3%)	13 (25.5%)	0.23	− 13	− 8
Alcohol	55 (80.9%)	51 (75.0%)	0.34	41 (80.0%)	43 (84.3%)	0.69	− 6	4
Marijuana and cannabis	8 (11.8%)	14 (20.6%)	0.18	6 (11.8%)	7 (13.7%)	1.00	9	2
Illegal drugs (cocaine, meth, opiates)	1 (1.5%)	1 (1.5%)	1.00	1 (2.0%)	1 (2.0%)	1.00	0	0
Prescribed opioids	23 (33.8%)	3 (4.4%)	< 0.01	25 (49.0%)	7 (13.7%)	< 0.01	− 29	− 35

### Correlations Between Employment and Health Outcomes

Finally, we examined the Pearson correlations between two-year employment outcomes (number of months worked and monthly earnings) and two-year change scores between baseline and two-year follow-up for five health measures, as shown in Table 9. The correlations were all in the predicted direction, that is, employment was positively associated with improved health outcomes. Three health outcomes significantly correlated with monthly earnings: improved mental health, reduced depression, and improved financial well-being.

**Table 9:** Pearson correlations between 24-month employment outcomes and change in health outcomes (baseline to 24 months)

Health measure	Months worked	Monthly earnings
SWLS^a^	0.18	0.17
PCS^b^	0.13	0.09
MCS^b^	0.17	0.23*
PHQ-9^c^	− 0.18	− 0.20*
IFDFW^d^	0.16	0.28**

^*^p < 0.05, **p < 0.01

^a^Satisfaction with Life Scale: 1 = low satisfaction – 7 = high satisfaction

^b^PCS = Physical Component Summary; MCS = Mental Component Summary Higher scores indicate better health

^c^Patient Health Questionnaire-9: 1–4 Minimal depression while 20–27 Severe depression

^d^InCharge Financial Distress Financial Wellbeing Scale: 1: High distress/Low wellbeing—10: Low distress/High well-being

## Discussion

This study evaluated two-year outcomes for NCCP, an intensive employment program featuring career coaching and financial assistance to veterans recently transitioning from the military. During the first year of follow-up, NCCP was more successful than LCR in engaging participants. Most NCCP participants used multiple components of the program and were satisfied with the help they received. Participants tapered their use of NCCP in the following year but continued to report satisfaction. By contrast, LCR participants used local community resources sparingly in the first year and even less in the second year.

Over a two-year follow-up period, NCCP achieved better outcomes than LCR on several employment measures. NCCP participants worked more months and earned more than LCR participants. Changes in health and well-being from baseline to 2-year follow-up demonstrated improvements in physical and mental health for NCCP participants in comparison to LCR participants.

The significant differences between NCCP and LCR over a two-year period were remarkably consistent with the one-year findings, but the employment outcomes became stronger over time. Over the two years, the total sample significantly increased the monthly employment rates, the percentage of months worked, and monthly earnings, but the employment outcomes during the second year were significantly better for NCCP than LCR.

The longitudinal findings, which show sustained employment outcomes favoring NCCP, differ from evaluations of employment models that provide short-term outcomes that dissipate once the intervention has ended (for example, as is true for transitional employment) (Penk et al., 2010). Instead, our study findings are similar to outcomes for evidence-based employment models, such as Individual Placement and Support, that have sustained or improved positive outcomes over time (Baller et al., 2020). NCCP participants’ continued contact with their career coaches during the second year may have been a contributing factor, although many participants tapered their rate of contact once they obtained employment. Competitive employment is self-reinforcing, and the greater improvements for the NCCP group may indicate that the program helped them to find more suitable jobs, ones that matched their interests and skills.

The robust, consistent employment outcomes in both groups over two years suggest that most participants became more securely attached to the labor force. At two years most were working full-time at a job they had held on average for 11 months or more—a remarkable finding in light of the sample’s high levels of service-related disability, depression, posttraumatic stress disorder, and general medical problems (Bond et al., 2022a). The successful employment outcomes for the control group created a ceiling effect, making it more difficult to show that NCCP had an impact. These successful outcomes for the LCR group are surprising compared to little or no improvement for participants receiving services as usual in most evaluations of employment services for people with disabilities.

The significantly better mental and physical health outcomes for the NCCP group are also unexpected. Prior studies of employment programs shown to be effective in improving employment outcomes have often failed to show a direct program impact on health outcomes (Frederick & VanderWeele, 2019; Wallstroem et al., 2021). Instead, successful employment leads to better health outcomes in many studies (Drake & Wallach, 2020; Modini et al., 2016; Waddell & Burton, 2006). The current study found positive associations between earnings and both mental health outcomes and financial well-being. While the correlational nature of these findings precludes any causal conclusions, the findings are consistent with numerous studies showing that employment is a social determinant of health.

Between baseline and two-year follow-up, the percentage of participants using tobacco, alcohol, and marijuana use did not change in either group. However, in both groups the percentage using prescribed opioids dramatically declined, suggesting that these medications are more frequently prescribed in the military than in civilian communities.

Taken together, the significantly better employment and health outcomes for NCCP support policy changes to expand access for veterans to effective employment programs such as NCCP, especially during the transition from military life. Because NCCP is a multi-faceted employment intervention, we do not know which component or components account for the better employment and health outcomes. Our ongoing research will examine this question.

Study limitations include no shows, sample attrition, and generalizability. Nine percent of participants randomized to the study did not participate in the research after the baseline interview, most of whom also likely did not participate in their assigned employment group. No shows reported significantly greater mental health symptoms than the final analytic sample. Because no shows were equally divided between NCCP and LCR, this limitation did not affect the internal validity, although it reduces the external validity. Future implementation of NCCP might consider program modifications, such as greater emphasis on health care, to increase engagement in employment services, because this group might especially benefit from NCCP, which improved both employment and mental health outcomes.

This study leaves unanswered questions of generalizability about both the population and intervention. Future research is needed to determine whether the study findings generalize to veterans with greater disabilities, older veterans, veterans who transition for a longer time period, and so forth. Regarding the intervention, the National Career Coach Program was possible through generous funding; how this employment could be implemented on a wide scale is unknown at this time.

During the first year of follow-up, interview completion rates were satisfactory, falling within the range of completion rates for follow-up studies using telephone interviews (Hendra & Hill, 2019). The attrition rate was higher in the second year, especially for the 24-month interview. Moreover, we found differential attrition for the control group, though the NCCP and LCR completer groups did not differ on baseline characteristics. Regarding generalizability, study participants consisted of a self-selected group who volunteered to enroll in the study after receiving a mail invitation or exposure to advertising in various media outlets. While the study inclusion criteria excluded many who may have participated, an unknown number of those meeting inclusion criteria declined participation. In sum, we do not know how broadly the study findings would apply to the general transitioning veteran population, including older veterans, those with even more serious mental health disorders, and those without any service-connected disabilities. Other limitations include the reliance on self-report and treatment contamination, that is, the fact that some participants received help from employment programs beyond those to which they were assigned. One additional study limitation was the use of single-item satisfaction measures for job satisfaction and satisfaction with services. In both cases, actual behavior is more credible than self-report; job satisfaction is associated with longer job tenure and satisfaction with services is associated with continued use of these services. The latter measures are more valid.

## Conclusions

This randomized controlled trial showed that the National Career Coach Program is effective in increasing employment outcomes and improving physical and mental health in veterans with significant mental health conditions and service-connected disability over a two-year period. Replications should assess the generalizability and durability of these findings. Given the alarming increase in adverse outcomes for transitioning veterans in the post-9/11 era, policy makers should consider increasing access to effective employment services targeting this at-risk population.

## Supplementary Information

Below is the link to the electronic supplementary material.

Supplementary file1 (DOCX 37 kb)
