# Supplementary material for: A Randomized Controlled Trial of an Employment Program for Veterans Transitioning from the Military: Two-Year Outcomes
Source: Adm Policy Ment Health. 2022 Jul 12;49(6):1072–83. doi: 10.1007/s10488-022-01208-z (PMC9616746; doi:10.1007/s10488-022-01208-z)
Supplement: Supplementary file 1 — Supplementary file1 (DOCX 37 kb) [file 10488_2022_1208_MOESM1_ESM.docx]

**Table A. Comparison of 24-Month Completers to No-Shows and Dropouts on Baseline Characteristics**

|  |  | | |  | |  |  | |  |  |
| --- | --- | --- | --- | --- | --- | --- | --- | --- | --- | --- |
| **Characteristic** | **24-Month Completers (N=125)** | | | **24-Month Dropouts (N=83)** | | **No-Shows (N=21)** | **Test of Significance** | |  |  |
| Female | 26 (20.8%) | | | 13 (15.7%) | | 5 (23.8%) | χ^2^=1.20, p=.55 | |  |  |
| Age, M (SD) | 31.22 (6.62) | | | 29.39 (6.92) | | 28.52 (5.54) | F=2.75, p=.07 | |  |  |
| **Marital status, *n* (%)** |  | | |  | |  |  | |  |  |
| Unmarried | 46 (36.8%) | | | 38 (45.8%) | | 14 (66.7%) | χ^2^=7.03, p=.03 | |  |  |
| Married | 79 (63.2%) | | | 45 (54.2%) | | 7 (33.3%) |  | |  |  |
| **Education, *n* (%)** |  | | |  | |  |  | |  |  |
| High school diploma/GED | 10 (8.0%) | | | 22 (26.5%) | | 2 (9.5%) | χ^2^=14.03, p=.001 | |  |  |
| Higher education (college, technical certificate) | 115 (92.0%) | | | 61 (73.5%) | | 19 (90.5%) |  | |  |  |
| **Race, *n* (%)** |  | | |  | |  |  | |  |  |
| White | 53 (42.4%) | | | 40 (48.2%) | | 13 (61.9%) | χ^2^=2.94, p=.23 | |  |  |
| Other | 72 (57.6%) | | | 43 (51.8%) | | 8 (38.1%) |  | |  |  |
| **Hispanic/Latino Ethnicity, *n* (%)** | 21 (16.8%) | | | 19 (22.9%) | | 4 (19.0%) | χ^2^=1.29, p=.52 | |  |  |
| **Military Branch, *n* (%)** |  | | | | | | | |  |  |
|  |  |  |  |  |  |  |  |  |  |  |
| Army | 83 (66.4%) | | | 49 (59.0%) | | 12 (57.1%) | χ2=1.49, p=.48 (Army vs. Other) | |  |  |
| Air Force | 18 (14.4%) | | | 10 (12.0%) | | 3 (14.3%) |  |  |  |  |
| Navy | 16 (12.8%) | | | 11 (13.3%) | | 3 (14.3%) |  |  |  |  |
| Marine Corp | 7 (5.6%) | | | 10 (12.0%) | | 3 (14.3%) |  |  |  |  |
| Coast Guard | 1 (0.8%) | | | 3 (3.6%) | | 0 (0.0%) |  |  |  |  |
| On Active Duty at Baseline | 40 (32.0%) | | | 16 (19.3%) | | 4 (19.0%) | χ2=4.79, p=.09 | |  |  |
|  |  | | |  | |  |  | |  |  |
| **Table A (continued). Comparison of 24-Month Completers to No-Shows and Dropouts on Baseline Characteristics** | | | | | | | | |  |  |
|  | | **24-Month Completers (N=125)** | **24-Month Dropouts (N=83)** | | **No Shows/ Withdrawals (N=21)** | | | **Test of Significance** | |  |
| Served in a combat zone, *n* (%) | 62 (49.6%) | | | 38 (45.8%) | | 8 (38.1%) | χ2=10.70, p=.03 | |  |  |
|  | 9.10 (7.04) | | | 8.10 (5.96) | | 7.61 (5.69) |  | |  |  |
| Years in service, M (SD) |  |  |  |  |  |  | F=.85, p=.43 | |  |  |
|  |  |  |  |  |  |  |  | |  |  |
| **Military Occupational Specialty, n (%)** | | | | | | | | |  |  |
|  |  |  |  |  |  |  |  |  |  |  |
| Administrative | 23 (18.4%) | | | 5 (6.0%) | | 3 (14.3%) | χ2=6.39, p=.04^a^ | |  |  |
| Combat Specialty | 13 (10.4%) | | | 12 (14.5%) | | 6 (28.6%) |  |  |  |  |
| Construction | 2 (1.6%) | | | 0 (0.0%) | | 0 (0.0%) |  |  |  |  |
| Electronic and Electrical Repair | 15 (12.0%) | | | 9 (10.8%) | | 2 (9.5%) |  |  |  |  |
| Engineering, Science, and Technical | 22 (17.6%) | | | 16 (19.3%) | | 3 (14.3%) |  |  |  |  |
| Healthcare | 9 (7.2%) | | | 6 (7.2%) | | 1 (4.8%) |  |  |  |  |
| Human Resource Development | 14 (11.2%) | | | 4 (4.8%) | | 1 (4.8%) |  |  |  |  |
| Machine Operator and Production | 3 (2.4%) | | | 1 (1.2%) | | 1 (4.8%) |  |  |  |  |
| Protective Service | 2 (1.6%) | | | 3 (3.6%) | | 1 (4.8%) |  |  |  |  |
| Support Service | 5 (4.0%) | | | 4 (4.8%) | | 1 (4.8%) |  |  |  |  |
| Transportation and Material Handling | 8 (6.4%) | | | 14 (16.9%) | | 1 (4.8%) |  |  |  |  |
| Vehicle and Machinery Mechanic | 9 (7.2%) | | | 9 (10.8%) | | 1 (4.8%) |  |  |  |  |
| ^a^ Combat and Protective Services Specialties compared with other Military Occupational Specialties | | | | | | | | |  |  |

**Table A (continued). Comparison of 24-Month Completers to No-Shows and Dropouts on Baseline Characteristics**

|  | | | | |  |
| --- | --- | --- | --- | --- | --- |
|  | **24-Month Completers (N=125)** | **24-Month Dropouts (N=83)** | **No Shows/ Withdrawals (N=21)** | **Test of Significance** |  |
| **Measures and Scales** | | | | |  |
| **Satisfaction with Life,^a^ M (SD)** | 4.65 (1.34) | 4.37 (1.40) | 3.68 (1.79) | F=4.59, p=.01 |  |
| **VR-12,^b^ M (SD)** | | | | |  |
| Physical Component Score (PCS) | 41.29 (11.16) | 39.46 (10.76) | 42.03 (9.15) | F=.89, p=41 |  |
| Mental Component Score (MCS) | 45.34 (13.28) | 41.15 (15.64) | 35.74 (15.19) | F=5.03, p=.01 |  |
| **PHQ-9,^c^ M (SD)** | 7.50 (5.79) | 9.67 (6.53) | 12.19 (6.37) | F=6.88, p=.001 |  |
| **IFDFW,^d^ M (SD)** | 5.78 (2.66) | 4.77 (2.25) | 4.70 (2.77) | F=4.69, p=.01 |  |
| **Substance Use** | | | | |  |
| **Tobacco, *n* (%)** | 40 (32.0%) | 34 (41.0%) | 12 (57.1%) | χ2=5.49, p=.06 |  |
| **Alcohol, *n* (%)** | 99 (79.2%) | 67 (80.7%) | 20 (95.2%) | χ2=3.05, p=.22 |  |
| **Marijuana/Cannabis, *n* (%)** | 14 (11.2%) | 16 (19.3%) | 2 (9.5%) | χ2=4.98, p=.29 |  |
| **Illegal Drugs, *n* (%)** | 2 (1.6%) | 2 (2.4%) | 1 (4.8%) | χ2=2.64, p=.62 |  |
| **Prescribed Opioids, n (%)** | 51 (40.8%) | 35 (42.2%) | 10 (47.6%) | χ2=.35, p=.84 |  |
| ^a^ 1 low satisfaction - 7 high satisfaction | |  |  |  |  |
| ^b^ Higher scores indicate better health | |  |  |  |  |
| ^c^ Scores range from Minimal (1-4) to Severe (20-27) | |  |  |  |  |
| ^d^ InCharge Financial Distress Financial Wellbeing Scale: Scores range from High (1.0) to Low (10.0) Distress | | | | |  |
